# The United States Veterinary Medical Association

**Published:** 1891-09

**Authors:** 


### THE UNITED STATES VETERINARY MEDICAL ASSOCIATION.

The 28th annual meeting of the United States Veterinary Medical Association, will be held at Willard's Hall, Willard's Hotel, Washington, D. C., on Sept. 15th to 17th 1891.

Arrangements have been made with all railroads in the Trunk Line Association, Southern Traffic Association, and Central Traffic Association, who will grant the usual one-third rates to all members and delegates in attendance. All persons attending this meeting will procure certificates from the railroad ticket agent at point of starting, paying full fare going, and present their certificates for the signature of the Secretary of the Association at the meeting, which will entitle them to a one-third rate returning. Tickets will be issued three days in advance of the meeting and will be good for three days after the meeting.

All members in or near Chicago are advised to join the party leaving Chicago on Sunday September 13, 1891, at 2:55 P. M., via the Baltimore and Ohio, and are advised to confer with Dr. W. L. Williams, Bloomington, Ill., at once in regard to arrangements.

The Program, beginning on Sept. 15th, will be as follows:

8:30 A. M., Meeting of the Comitia Minora; 10 A. M., Annual meeting convened; Roll Call; Address of welcome to the members by the President; Unfinished Business; Reception and consideration of reports of the Comitia Minora.

At 12:30 noon adjournment for lunch, provided by the courtesy of the Veterinarians of Washington and Baltimore.

Afternoon Session:—Reports of Committees.

First, Committee on Intelligence and Education; second, Finance Committee; third, Committee on Diseases; fourth, Prize Committee; fifth, Special College Committee; sixth, Committee on Army Legislation; seventh, Publication Committee; eighth, Special Committee on a Central Organized Body; ninth, Special Committee on food Inspection; tenth, Secretary's Report; eleventh, Reports from Assistant (State Foreign) Secretaries; Discussion of reports of Committees; Election of Officers; New Business.

Sept. 16th 1891, Morning Session:—

10 A. M. Roll Call; Papers by Dr. C. C. Lyford, Minneapolis, Minn., on "Barren Mares"; by Dr. W. Bryden, Boston, Mass., on "Foreign Cattle Transportation, and its Regulations"; by Dr. W. L. Williams, Bloomington, Ill., on "Rachitis"; by Dr. R. S. Huidekoper, New York, on "Identification." Postponed discussion on Prof. Liautard's paper on "Veterinary Jurisprudence." Several other papers of interest will be offered.

Discussion of Papers and Reports of Cases. At 7 P. M., a banquet will be held at Willard's Hotel.

The headquarters of the Association will be at Willard's Hotel, where a special rate of $3.00 per day has been made for members.

It is specially important that, immediately upon arrival, the members will report at the Reception room and register, giving full particulars as to city address, etc.

This meeting promises to have the largest attendance of any since the organization, in 1863, and every member should attend, and do his share towards its success.

Committee of Arrangements.—Dr. R. S. Huidekoper, Chairman, New York; Dr. W. Horace Hoskins, Philadelphia; Dr. Wm. Dougherty, Baltimore.

Local Committee of Arrangements:—Dr. E. S. Walmer, 3222 M. St., Dr. F. L. Kilborne, Dr. A. A Swedburg, Washington, D. C., Drs. Wm. Dougherty, G. C. Faville, A. W. Clement, and W. H. Martinet of Baltimore.

